# Evaluation of Stress Trajectories in Craniofacial Bones in Class I Skeletal Pattern: A Finite Element Analysis Using CT Scan

**DOI:** 10.7759/cureus.106868

**Published:** 2026-04-11

**Authors:** Shruthi D P, Amitabh Kallury, Nagaraj B M, Shahana S

**Affiliations:** 1 Orthodontics and Dentofacial Orthopedics, Government Dental College and Research Institute, Bengaluru, IND; 2 Orthodontics and Dentofacial Orthopedics, People's College of Dental Sciences and Research Centre, Bhopal, IND; 3 Pharmacology, Shridevi Institute of Medical Sciences and Research Hospital, Tumkur, IND

**Keywords:** cranial base, finite element analysis, occlusal forces, orthodontics, stress trajectories

## Abstract

Background: Occlusal forces are transmitted through the craniofacial skeleton, but the extent to which these stresses propagate to the cranial base remains unclear. Finite element analysis offers a precise tool to study stress distribution in complex craniofacial structures.

Aim of the study: To evaluate stress trajectories generated by occlusal loading and determine their extension to the cranial base using CT-derived finite element models.

Setting and design: An in-silico finite element study was conducted at the Department of Orthodontics and Dentofacial Orthopedics, Government Dental College & Research Institute, Bengaluru, India.

Methods and materials: CT scans of healthy adults with Class I skeletal and dental patterns were converted into three-dimensional (3D) finite element models. Occlusal loads of 1000 N, 500 N, and 350 N were applied to both jaws and to the mandible alone. Stress distribution was assessed across craniofacial structures.

Statistical analysis: Descriptive analysis of stress magnitudes and distribution patterns; no inferential statistics were applied.

Results: High occlusal loads (1000 N, 500 N) generated stresses in the mandible exceeding cortical bone yield limits (>100 MPa), while cranial base stresses remained negligible (~0.5 MPa). Physiological loading (350 N) produced mandibular stresses within cortical bone tolerance (~76 MPa). Masseter stresses were higher in combined maxilla-mandible loading compared to mandibular-only loading.

Conclusions: Occlusal forces are primarily dissipated within the mandible and maxilla, with minimal extension to the cranial base. Finite element analysis confirms the biomechanical safety of orthodontic loading with respect to cranial structures.

## Introduction

Bone is a dynamic tissue that is normally renewed through balanced bone resorption and formation processes that are choreographed in space and time. It is remodelled to meet its mechanical demands, suggesting that mechanical forces are among the most potent factors that influence bone formation and resorption [1].

During mastication, the occlusal forces are transferred from the teeth through the periodontal ligaments, mandible, maxilla, and temporomandibular joint, up to the cranial base. According to the photoelastic analysis and finite element analysis, there are three main stress trajectories in the face: the maxillonasal, maxillozygomatic, and maxillopterygoid stress trajectories. Therefore, the occlusal forces loaded onto the maxillary teeth could be distributed to all the facial elements with no significant stress concentration on the alveolar bone [2].

The use of the finite element method allows studying a single tooth, a set of teeth, or even the relationship between maxillary and mandibular dental arches on a more solid and precise biomechanical basis than other methods, such as photoelastic models and strain gauges. Therefore, with this methodology, it is possible to have quantitative and qualitative representations of dental and mandibular biomechanics to evaluate displacements, strains, and stresses, which may occur in biomechanical structures [3].

Although few studies in the past have evaluated the stress trajectories in maximum intercuspation in isolation, no attempt was made to evaluate comprehensively the stress trajectories of all structures involved in mastication up to the base of the cranium. Also, the earlier methods of evaluation involved the use of either conventional methods or the cone beam CT (CBCT) approach. The current in-silico study aims to evaluate the stress trajectories using CT scans. This approach may bring in a holistic understanding of stress trajectories up to the cranium level and may give additional insights into the stomatognathic systems.

## Materials and methods

The study protocol was reviewed and approved by the Institutional Review Board of the Government Dental College & Research Institute, Bengaluru, India (approval no. GDCRI/IEC-ACM(2)/01/2023-24). All procedures were planned in accordance with the ethical standards of the responsible committee on human experimentation and with the Helsinki Declaration (1975, revised 2000). Written informed consent was obtained from the participant prior to enrolment.

This is an in-silico study conducted over a period of one year at the Department of Orthodontics and Dentofacial Orthopedics, Government Dental College & Research Institute, Bengaluru, India.

Selection criteria

Inclusion Criteria

The study included one healthy adult (>18 years) with a Class I skeletal and dental pattern; third molars may or may not be erupted without a history of trauma or fracture to the craniofacial region.

Exclusion Criteria

The exclusion criteria were as follows: age <18 years; syndromic conditions; bruxism; missing/supernumerary/decayed teeth, prostheses, or implants; prior orthodontic treatment; periodontal or temporomandibular joint disease; neuralgias; any systemic medical conditions contraindicating CT scans.

Subjects meeting the inclusion criteria were planned to undergo CT scanning. CT data were obtained using a multi-slice CT scanner. Scanning was performed with a slice thickness of 0.5-1 mm, tube voltage of 120 kVp, and tube current of 100-200 mA. The images were acquired in DICOM format with a matrix size of 512 × 512 pixels and a field of view adjusted to include the entire craniofacial region.

The anatomical data obtained in DICOM format were imported into 3D Slicer software (Slicer Community, Brigham and Women’s Hospital, Boston, MA, USA) to generate three-dimensional (3D) models of individual teeth, maxilla, mandible, and temporomandibular joint up to the cranial base. The 3D models were saved in STL format for further processing.

Methodology

Conversion of DICOM Model Into a Finite Element Model

The output data from the 3D slicer software was then exported to the Hypermesh 22.0 program (Altair Engineering Inc., Troy, MI, USA), which was then used to create a finite element model of the skull with all its complexities.

Meshing/Discretization

The finite element modeling was performed using second-order tetrahedral elements for discretizing the domain, where the tetrahedral elements were used to capture all the complexities of the human skull. The finite element model thus created had a total of 1257159 nodes and 5397932 elements, respectively.

Convergence Checks and Model Validation

A minimum of three progressively refined meshes were generated and are listed in Table 1. The characteristic element length used in the model was 0.5 mm.

**Table 1:** Meshes and elements

Mesh Level	Total Elements	Purpose
Coarse	0.5	Baseline discretization
Medium	0.3	Intermediate resolution
Fine	0.1	Reference solution

Interpretation of Convergence Behaviour

The convergence trends revealed that global responses (stress and deformation) converged rapidly with mesh refinement. Local quantities (stress) exhibited higher mesh sensitivity but stabilized beyond the medium-to-fine discretization. No qualitative change in deformation mode, failure pattern, or contact behavior was observed after the converged mesh level.

These observations indicate that the selected mesh density captures both global structural response and local nonlinear effects with sufficient fidelity.

Materials Used

The material properties used for the model are listed in Table 2.

**Table 2:** Material properties used in finite element model

Material	Young's Modulus (Mpa)	Poisson’s Ratio	Mass Density (tonne/mm^3^)
Cancellous bone	2000	0.3	8e^-10^
Cortical bone	20000	0.3	1.85e^-9^
Enamel	18600	0.31	1.9e^-9^
Periodontal ligament	1750	0.35	1.212e^-9^
Root	18600	0.33	2.1e^-9^
Medial pterygoid	0.8	0.45	1.16e^-9^
Lateral pterygoid	0.8	0.45	1.16e^-9^
Masseter	0.8	0.45	1.16e^-9^
Temporalis	300	0.45	6.6e^-10^
Skull	13500	0.22	1.8e^-9^

All materials were assumed to be isotropic and linearly elastic. Although craniofacial tissues exhibit anisotropic and nonlinear behavior in vivo, these simplifications are commonly adopted in finite element studies to reduce computational complexity and enable stable convergence.

Loading and Boundary Conditions

For the finite element model, varying occlusal load magnitudes were applied to the same anatomical regions. Six loading scenarios were evaluated.

In Case 1a, full-arch loading of 1000 N was applied independently to both the maxilla and mandible, with force distribution of 296 N at the canines, 224 N at the incisors, 400 N at the premolars, 520 N at the first molars, and 560 N at the second molars. In Case 1b, a total load of 1000 N was applied only to the mandibular region, with corresponding forces of 148 N, 112 N, 200 N, 260 N, and 280 N at the canines, incisors, premolars, first molars, and second molars, respectively.

In Case 2a, full-arch loading of 500 N was applied to both arches, distributing 148 N to the canines, 56 N to the incisors, 200 N to the premolars, 260 N to the first molars, and 280 N to the second molars, while Case 2b involved mandibular-only loading of 500 N with forces of 74 N, 28 N, 100 N, 130 N, and 140 N across the same tooth groups.

In Case 3a, full-arch loading of 350 N was applied independently to both arches, with forces of 103.6 N at the canines, 39.2 N at the incisors, 140 N at the premolars, 182 N at the first molars, and 196 N at the second molars, whereas in Case 3b, a 350 N load was applied only to the mandible with reduced forces of 51.8 N, 19.6 N, 70 N, 91 N, and 98 N, respectively.

**Figure 1:**
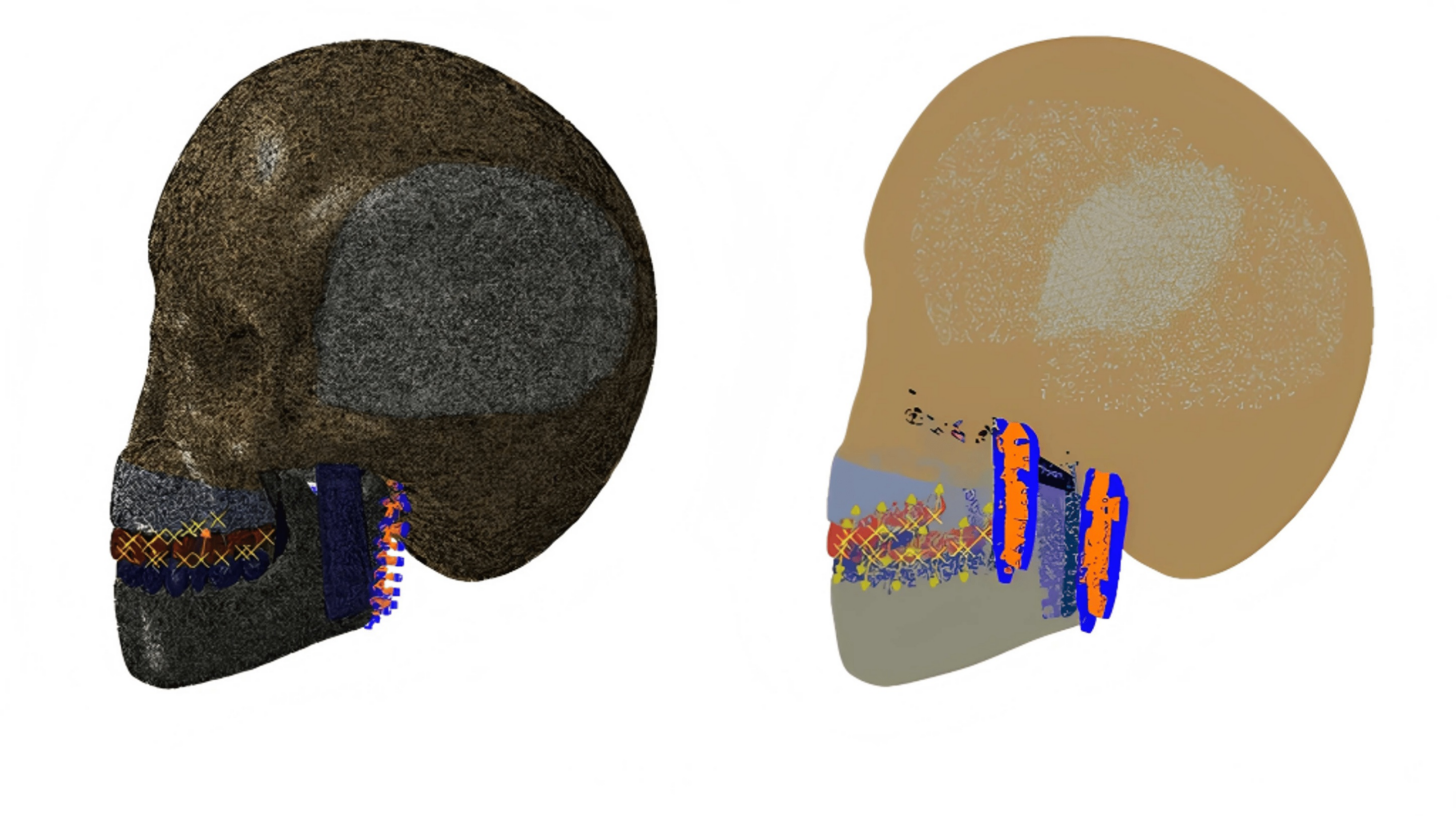
Three-dimensional finite element model of the skull with loading. This figure has been created using ABAQUS, version 2020 (Dassault Systèmes, Vélizy-Villacoublay, France).

In all the above cases, the model is constrained completely to the posterior margin of the ramus of the mandible. Figure 1 shows the 3D finite element model of the skull with the applied loading conditions.

## Results

Finite element analysis showed that the stress distribution pattern depended strongly on both the amount and site of occlusal loading. To represent different functional situations, three loading conditions were tested: high (1000 N), moderate (500 N), and physiological (350 N). Each load was first applied simultaneously to both the maxilla and mandible, and then to the mandible alone, to compare how stresses were transmitted through the craniofacial structures.

High load (1000 N)

**Figure 2:**
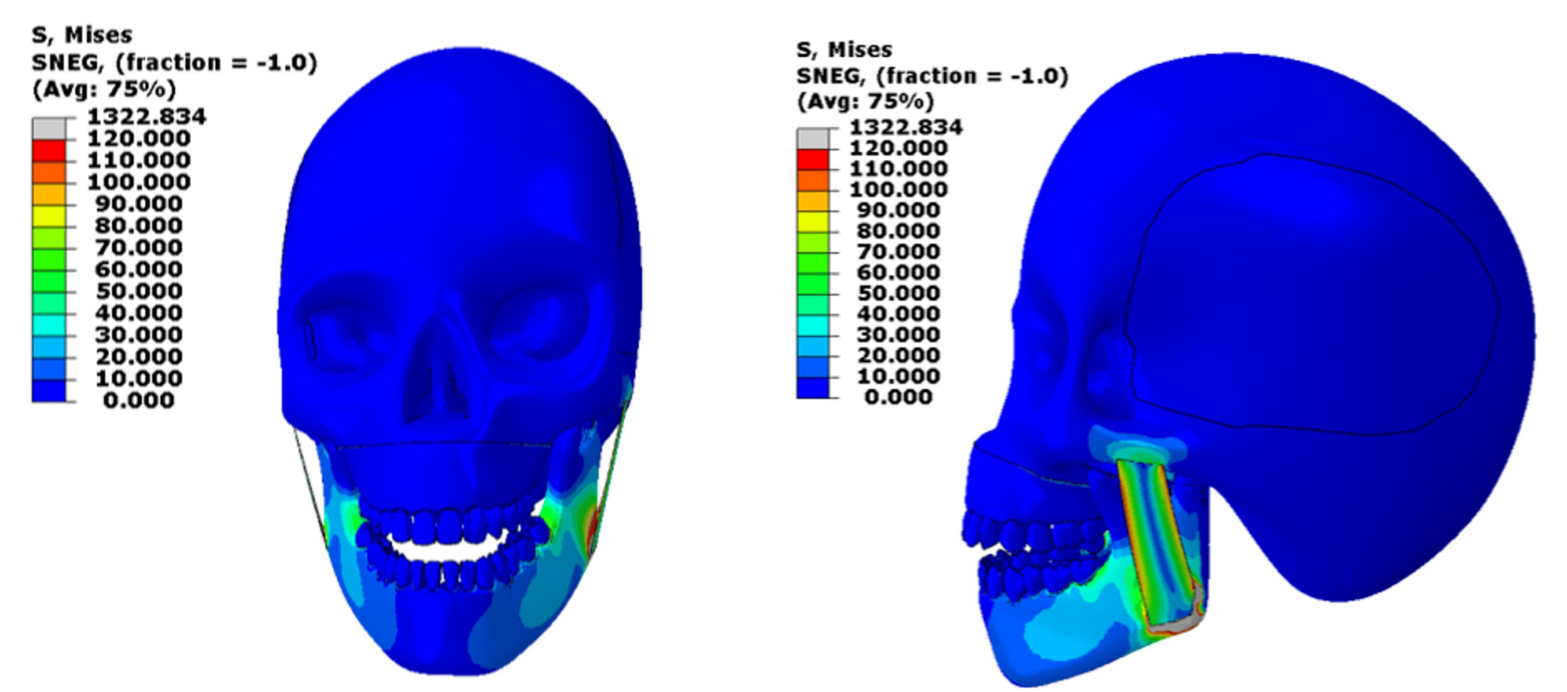
Von-Mises stress contour for the 1000N loading on the upper and lower jaws This figure has been created using ABAQUS, version 2020 (Dassault Systèmes, Vélizy-Villacoublay, France).

When a load of 1000 N was applied to both the upper and lower jaws together, the mandible showed very high stresses exceeding 230 MPa, which is far greater than the normal cortical bone yield range (50-150 MPa) [4]. The lower teeth, especially the lower second molar, are experiencing higher stress levels, exceeding the yield stress of the material. The masseter muscle also showed stresses beyond its physiological limit (0.2-0.4 MPa during contraction), indicating muscle overloading [5]. In contrast, stresses in the cranial base were negligible (~0.5 MPa), meaning that only minimal force was transmitted upward through the skull (normally around 75 to 136 MPa for young adults) [6]. Figure 2 shows the Von-Mises stress contour for the 1000N loading at the upper and lower jaw.

**Figure 3:**
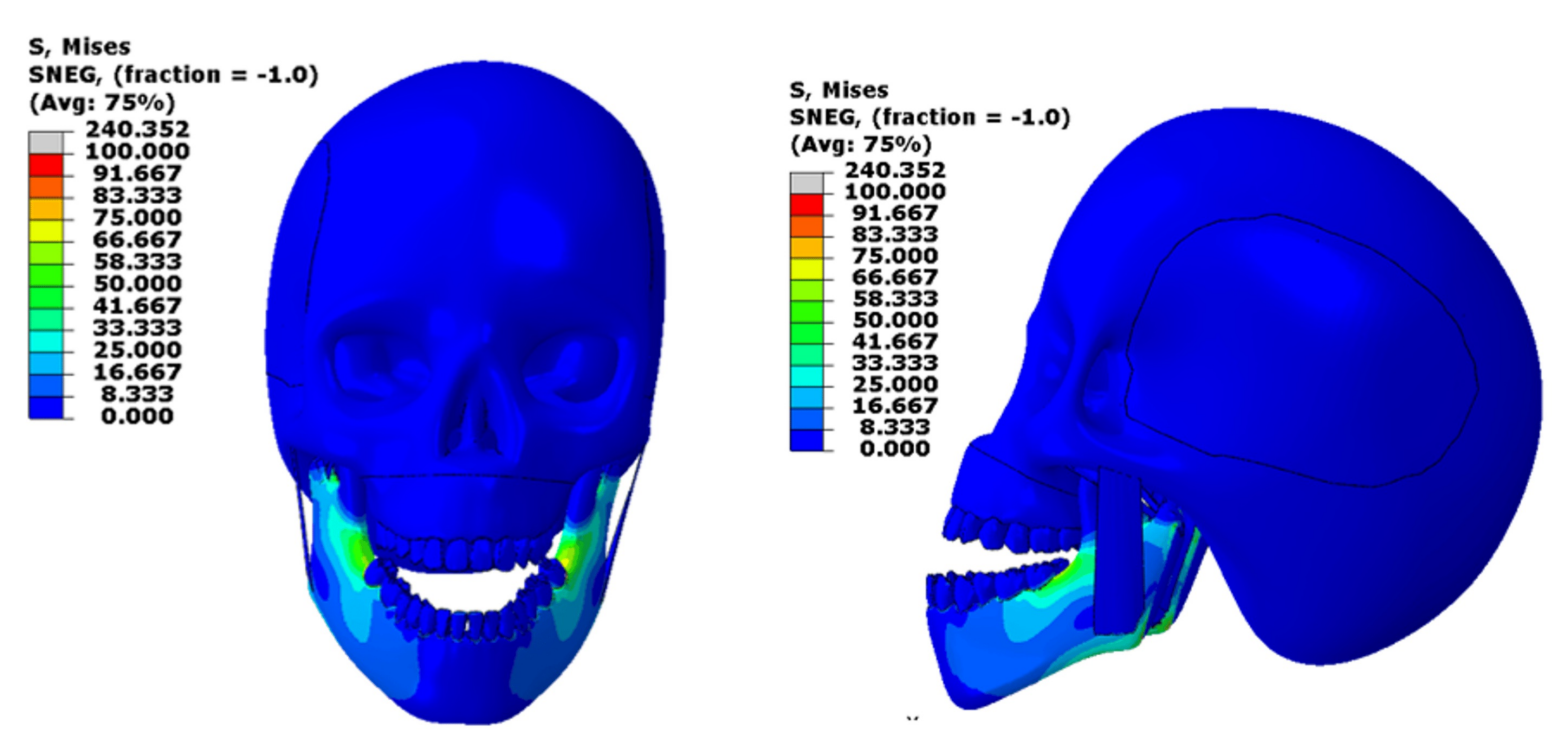
Von-Mises stress contour for the 1000N loading on the mandible only This figure has been created using ABAQUS, version 2020 (Dassault Systèmes, Vélizy-Villacoublay, France).

When the same 1000 N load was applied to the mandible alone, stress became concentrated mainly along the mandibular body and alveolar crest, with local peaks above 100 MPa, still higher than safe physiological limits for cortical bone (50-150 MPa) [4]. The masseter muscle is not stressed much. It is very much below the yield stress. The skull experienced almost no stress transmission, indicating that isolated mandibular loading increases local strain. Figure 3 shows the Von Mises stress contour for the 1000N loading on the mandible only. 

Moderate load (500 N)

**Figure 4:**
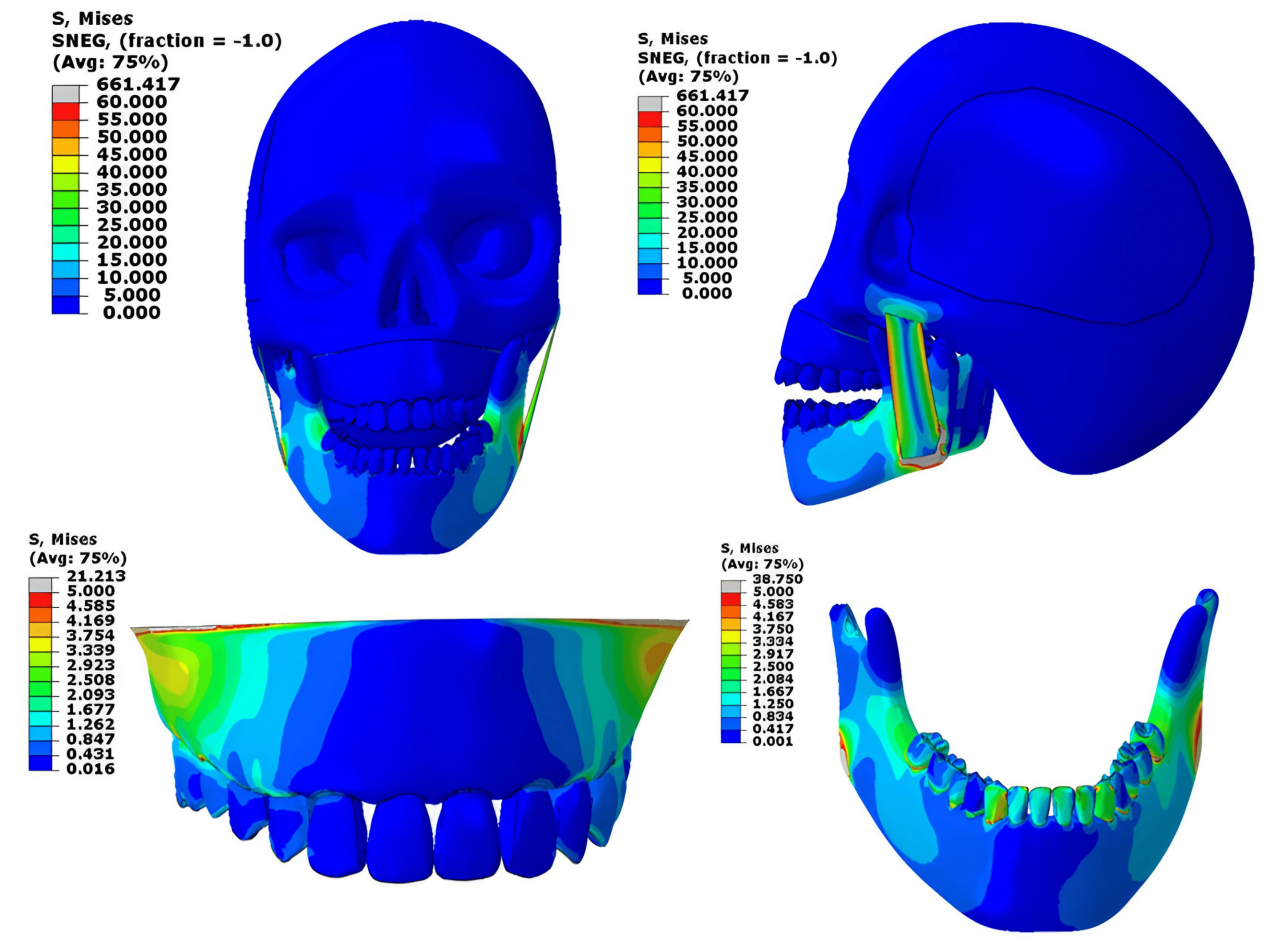
Von-Mises stress contour for the 500N loading at the upper and lower jaw This figure has been created using ABAQUS, version 2020 (Dassault Systèmes, Vélizy-Villacoublay, France).

With a 500 N load, stress was mainly concentrated at the mandibular molar area, reaching about 75 MPa. This is close to or slightly above the upper limit of cortical bone’s physiological tolerance (50-150 MPa) [4]. The lower teeth, especially the lower second molar, are experiencing higher stress levels, exceeding the yield stress of the material. The masseter muscle again showed higher-than-normal stress, suggesting that moderate clenching or chewing can still produce significant muscle strain (normally around 0.2-0.4 MPa during contraction) [5]. Stresses at the cranial base remained very low (normally around 75 to 136 MPa for young adults) (Figure 4).

**Figure 5:**
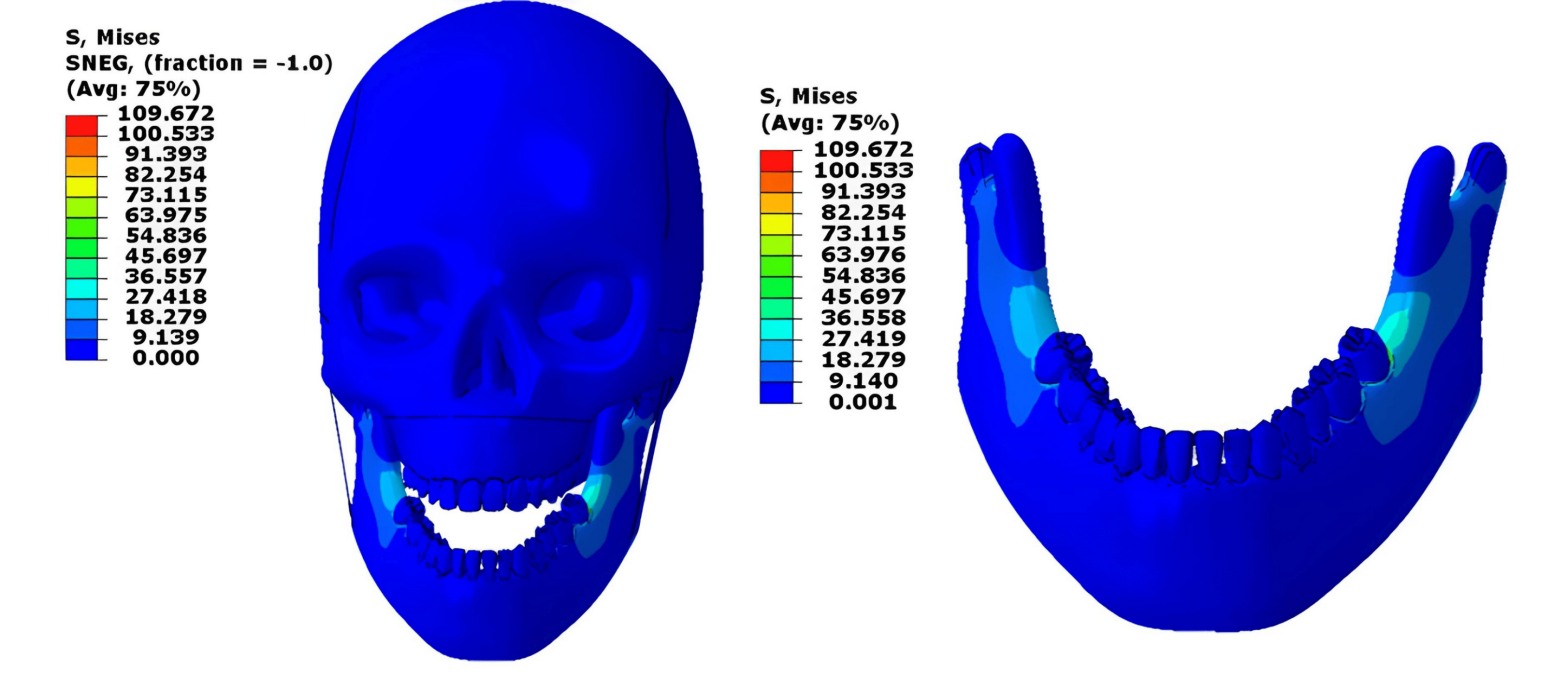
Von-Mises stress contour for the 500N loading on the mandible only This figure has been created using ABAQUS, version 2020 (Dassault Systèmes, Vélizy-Villacoublay, France).

The load was applied only to the mandible; peak stress increased to about 109 MPa, exceeding normal limits in some areas (50-150 MPa) [4], especially near the molars and mandibular angle. However, most of the bone remained within a safe range. The masseter and cranial base showed only mild stress levels, suggesting that the mandible itself absorbs most of the applied load (Figure 5). 

Physiological load (350 N)

**Figure 6:**
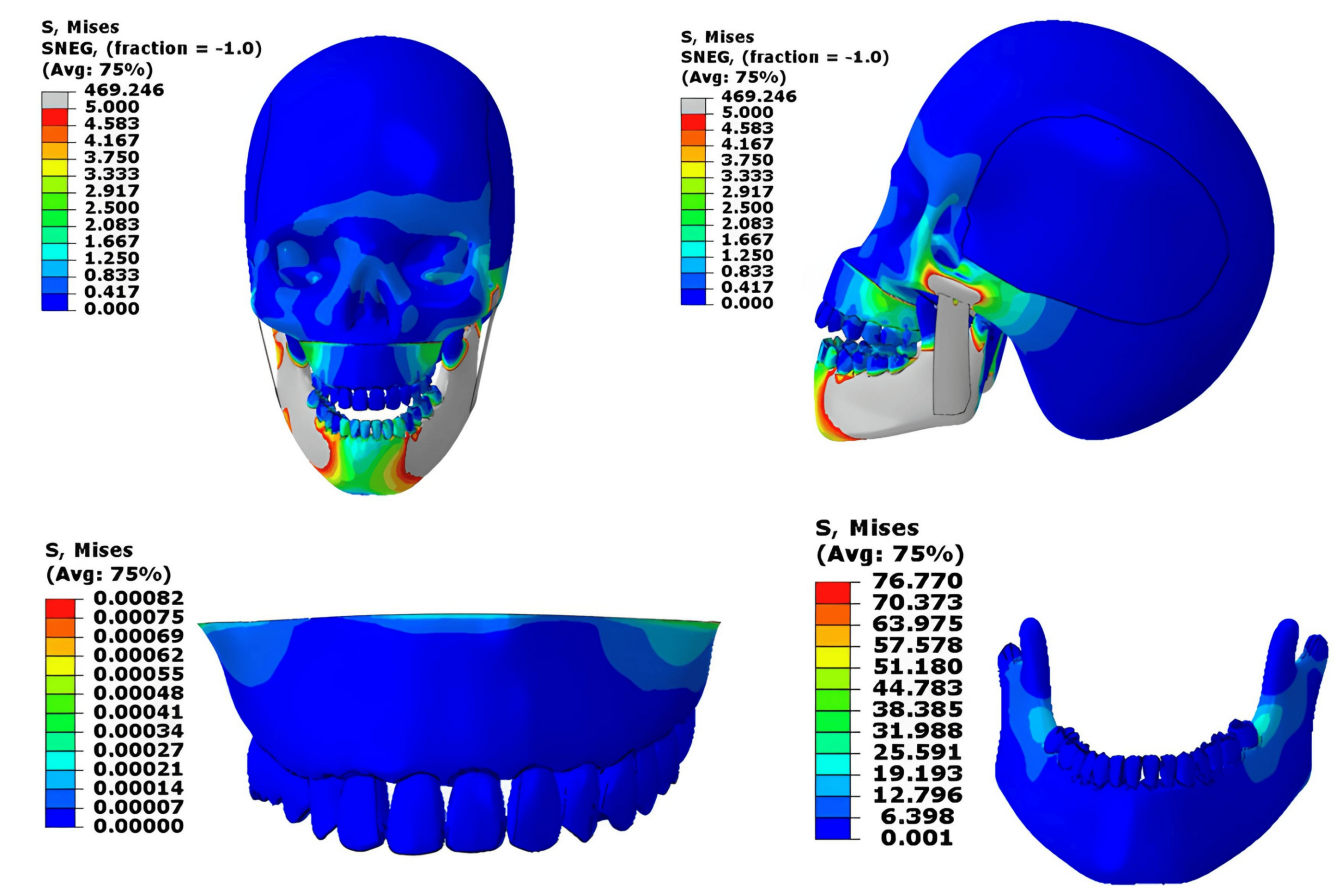
Von-Mises stress contour for the 350 N loading at the upper and lower jaw This figure has been created using ABAQUS, version 2020 (Dassault Systèmes, Vélizy-Villacoublay, France).

At 350 N, which represents normal chewing or biting force, stresses were mainly observed near the mandibular second molar, which is within the physiological range for cortical bone, indicating a safe and balanced distribution of load [4]. The lower teeth, especially the lower second molar, are experiencing higher stress levels, exceeding the yield stress of the material. The masseter muscle still showed slightly higher values than its normal functional range, reflecting the muscle’s active role in stabilizing the mandible during normal biting (normally around 0.2-0.4 MPa during contraction) [5]. Cranial base stresses were negligible, showing efficient absorption of physiological forces. (Figure 6)

**Figure 7:**
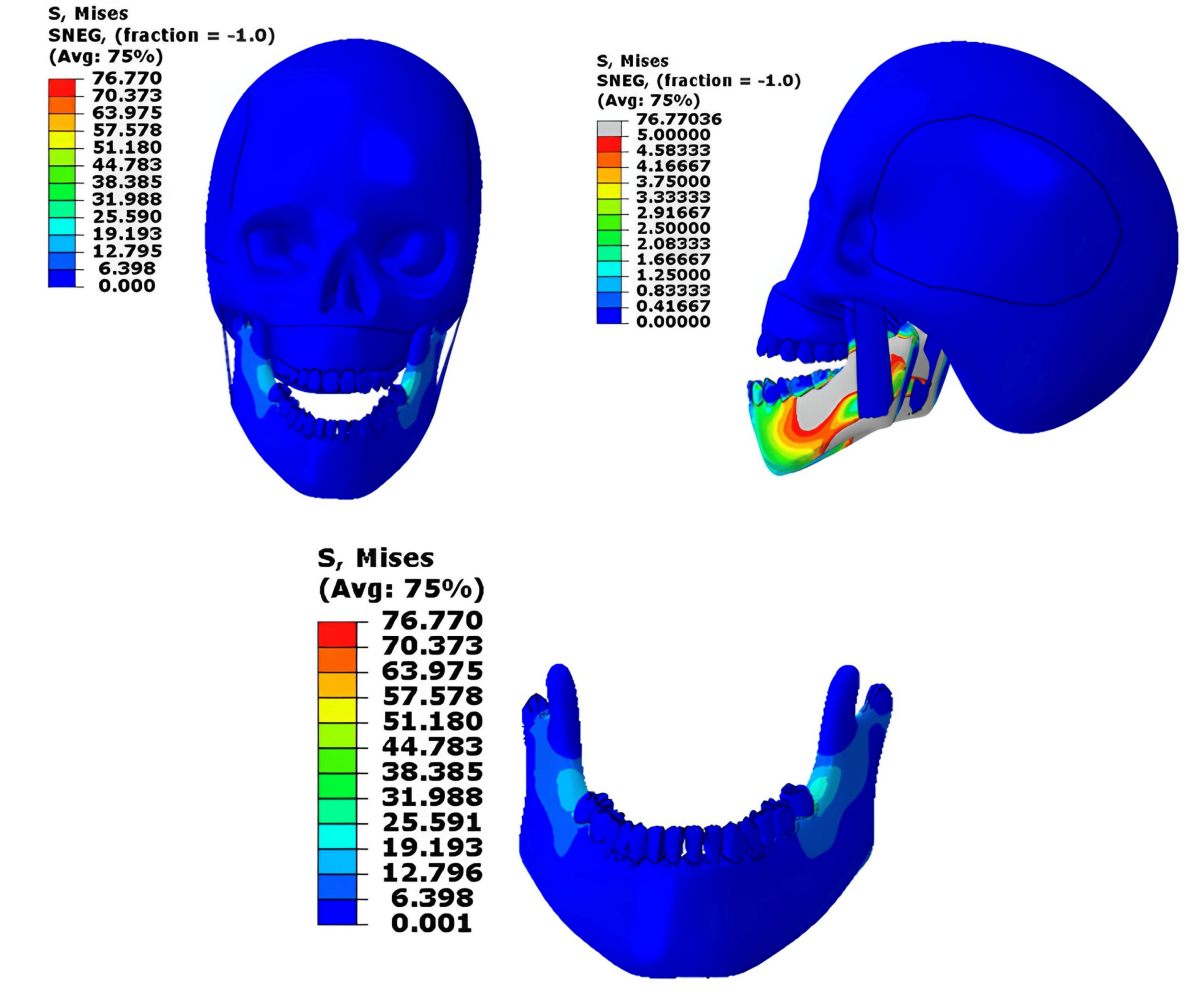
Von-Mises stress contour for the 350 N loading on the mandible only This figure has been created using ABAQUS, version 2020 (Dassault Systèmes, Vélizy-Villacoublay, France).

When the physiological load was applied only to the mandible, the pattern was very similar, with stresses concentrated in the same regions and peak values remaining within safe limits. The masseter and cranial base stresses remained low, confirming that under normal functional loading, the mandible experiences safe and physiologically acceptable stress levels (Figure 7).

## Discussion

The aim of the present study was to evaluate whether stress trajectories generated by occlusal loading extend to the cranial base, using finite element analysis of the human skull under varying occlusal forces. In this study, occlusal loading forces were first applied simultaneously to both the maxilla and mandible and subsequently applied separately to the mandible to evaluate stress distribution patterns. The results showed that the stresses were concentrated more in the mandibular region, while the cranial base exhibited negligible stress transmission across all loading conditions.

In this study, finite element analysis was employed in order to evaluate the stress distribution within the craniofacial skeleton under varying occlusal loads. Finite element analysis is a powerful tool for studying biomechanical changes in complex skeletal structures. In dentistry and orthodontics, it has been widely used to evaluate stresses and strains in oral and dental tissues under functional loading, overcoming the limitations of earlier experimental approaches like photoelasticity. Since its introduction to dental research, finite element analysis has become the preferred approach for investigating a wide range of biomechanical problems [7,8].

Finite element analysis was performed under three occlusal loading conditions (1000 N, 500 N, and 350 N), applied either to both jaws or to the mandible alone. A physiological biting force of approximately 350 N was considered, based on the findings of Proffit et al. (1983) [9,10]. At 350 N, stresses within the mandible remained largely within the yield stress limits of cortical bone (≤76 MPa), indicating a more physiological load distribution. At 1000 N and 500 N, stress values in the mandible exceeded the yield stress of cortical bone, while skull stresses remained negligible (~0.5 MPa). This suggests that the craniofacial skeleton effectively dissipates occlusal force locally, preventing its propagation into cranial base structures. Another finding observed was that, when the force was applied only to the mandible under all three loading conditions, high local stress concentrations were observed; however, masticatory muscles exhibited minimal stress compared to the both-jaw loading condition.

Gross et al. (2001) [11] used 3D finite element analysis to study the human facial skeleton under simulated occlusal loading. It was observed that full-arch loading in the maxilla produced a more uniform distribution of stresses, while single-point loading generated localized ‘V-shaped’ stress trajectories, particularly in the anterior region. It was concluded that the facial skeleton behaves like a vertical plate, efficiently dissipating occlusal forces through in-plane bending. This is consistent with the present study's findings of localized stress absorption in jaw structures and minimal cranial base involvement.

The present study was conducted to evaluate the extent of stress distribution to the cranial base during orthodontic treatment using finite element analysis. The results demonstrated that the stresses transmitted to the cranial base were negligible, suggesting that orthodontic forces are largely confined to the dentoalveolar and adjacent skeletal structures without significant cranial involvement. These findings reinforce the reliability of FEA as a valuable research tool, enabling precise visualization and quantification of stress patterns. Overall, the study highlights the biomechanical safety of applied orthodontic mechanics with respect to cranial base structures and emphasizes the utility of computational modeling in orthodontic research.

The limitation of this study is that, although CT-derived finite element modeling was used to approximate patient anatomy, it still represents a simulation. Biological variability, dynamic tissue responses, and clinical conditions may differ from the simulated environment, which could influence the actual stress distribution. As this study involves a single patient, the results observed should be interpreted as case-specific rather than broadly representative. While the model provides useful insights into biomechanical behavior under controlled loading conditions, caution is warranted in extrapolating these findings directly to clinical practice. Further studies incorporating multiple subjects and varied anatomical parameters are needed to improve external validity and support more generalized conclusions.

## Conclusions

Within the limitations of this finite element analysis study, it can be concluded that occlusal forces are predominantly absorbed and dissipated within the mandible and maxilla, with minimal transmission to the cranial base. This indicates that the craniofacial skeleton possesses an inherent biomechanical design that effectively localizes functional stresses within the dentoalveolar region. Even at higher occlusal loads, such as 1000 N and 500 N, stress propagation to the cranial base remained negligible, while the mandible experienced the greatest concentration of stresses. Under physiological loading of 350 N, the stress values were well within the yield limits of cortical bone, suggesting a safe and balanced distribution of masticatory forces. These results reaffirm that normal occlusal and orthodontic forces are biomechanically safe for cranial structures and are primarily confined to the jaws and associated skeletal regions.

Furthermore, the study highlights the significance of craniofacial buttresses and mandibular morphology in dissipating occlusal loads efficiently. The CT-derived finite element modeling approach proved valuable for visualizing stress trajectories and understanding how different loading conditions affect craniofacial biomechanics. Such insights can enhance clinical decision-making in orthodontics by ensuring that applied forces remain within physiological limits. Future investigations incorporating dynamic modeling, varied skeletal patterns, and patient-specific geometries could refine these findings and extend their clinical applicability. Overall, the present study underscores the role of finite element analysis as a reliable and precise tool for evaluating the biomechanical behavior of craniofacial structures under functional loads.
